# Epigenetic aging: insights from network biology

**DOI:** 10.18632/aging.100610

**Published:** 2013-10-20

**Authors:** Andrew E. Teschendorff

**Affiliations:** Statistical Cancer Genomics, UCL Cancer Institute; Centre for Mathematics and Physics in the Life Sciences and Experimental Biology, University College London, London WC1E 6BT, UK; CAS-Max Planck Partner Institute for Computational Biology, Shanghai Institute for Biological Sciences, Chinese Academy of Sciences, Shanghai 200031, China

While a number of key signalling pathways (e.g. mTOR signalling) and biological processes (e.g. telomere attrition) affecting lifespan have been identified [1,2], other theories have argued that aging results mainly from accumulated molecular damage [1]. Most likely, aging is determined by a complex cross-talk between multiple biological effects. Molecular damage itself can take many forms, including somatic DNA mutations and copy-number changes. The advent of novel biotechnologies, allowing routine genome-wide quantitative measurement of epigenetic marks, specially DNA methylation, have recently demonstrated that age-associated changes in DNA methylation, a phenomenon now known as “epigenetic drift”, may play an equally important role in contributing to the aging phenotype [3,4]. Indeed, like telomere attrition, epigenetic drift has been associated with stem cell dysfunction [5], disease risk factors and common age-related diseases, such as cancer and Alzheimer's [3]. Apart from extensive experimental work supporting a role for DNA methylation in aging, computational network biology approaches have recently shed further light into the potential role of epigenetic drift. For instance, one study has shown that drift appears to target WNT signalling, a key pathway in stem-cell differentiation and already known to be deregulated with age [3]. A more recent study mapped epigenetic drift occurring in gene promoters onto a human protein interactome and observed that most of the changes happen at genes which occupy peripheral network positions, i.e. those of relatively low connectivity [6]. Although developmental transcription factors make up a significant proportion of “drift genes”, the observed topological effect was not entirely driven by this enrichment. Crucially, the topological properties of genes undergoing epigenetic drift were highly distinctive from those which have been associated with modulating longevity, those undergoing age-related changes in expression, or those somatically mutated in age-related diseases like cancer (Figure 1). Moreover, essential housekeeping genes, many of which occupy highly central positions in the interactome, appear protected from epigenetic drift. Although the overall functional significance of epigenetic drift remains to be established, a few instances of epigenetic drift causing silencing of key transcription factors have already been reported [5]. Thus, it is plausible that epigenetic drift may gradually affect differentiation programs through functional deregulation of key lineage determining transcription factors, leading to well-known observations such as myeloid skewing of the aged hematopoietic system [5]. Epigenetic drift affecting key transcription factors may further increase predisposition to age-related diseases like cancer, by locking stem cells into states of self-renewal, and causing tissues to exhibit an increased cellular plasticity and diversity, a likely prerequisite for neoplastic formation [3]. The distinctive network topology of epigenetic drift is striking for another reason. A number of recent studies have observed that epigenetic drift kicks in straight after birth and appears to be particularly prominent during pre-puberty [3,7]. Thus, in stark contrast to age-related gene expression and cancer related mutational changes, which affect mainly genes of relatively high connectivity and which have been inferred from adult populations, epigenetic drift occurs throughout life. It is therefore plausible that if functional changes caused by epigenetic drift, occurring prior to and during reproductive age, were to affect essential, highly connected genes in the network that these would be weeded out by natural selection. If true, this could explain the enrichment of epigenetic drift at peripheral network positions and is reminiscent of the observation made by Goh et al that most non-essential heritable disease genes occupy peripheral network positions, since the associated heritable mutations are less likely to compromise viability [8]. In conclusion, given that a number of other studies have also indicated that the epigenome is particularly sensitive to environmental factors (e.g. nutrient deprivation) during early life, it could well be that epigenetic drift plays a key role, not only in determining the biological age of an individual, but also in evolution.

**Figure 1:**
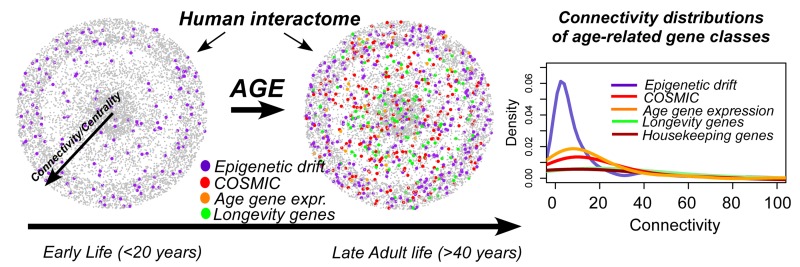
Epigenetic drift occurs throughout life, including pediatric populations and pre-puberty, targeting genes which occupy peripheral positions in the human interactome. Interactions have been suppressed and connectivity is approximated by the radial distance from the center. Genes undergoing age-related changes in expression, genes undergoing somatic mutations in cancer (COSMIC), longevity associated genes and housekeeping genes all exhibit widely different connectivity distributions, compared to genes undergoing epigenetic drift, as indicated on the right. Epigenetic drift targets many transcription factors bivalently marked in human embryonic stem cells, which co-locate at peripheral network positions. Thus, epigenetic drift may underlie the observed decline of stem cell function with age, immunosenescence, and may result in increased disease predisposition(e.g. cancer).

## References

[R1] Blagosklonny MV (2012). Aging.

[R2] Blackburn EH (2006). Nat Med.

[R3] Teschendorff AE (2013). Hum Mol Genet.

[R4] Heyn H (2012). Proc Natl Acad Sci U S A.

[R5] Beerman I (2013). Cell Stem Cell.

[R6] West J (2013). Proc Natl Acad Sci U S A.

[R7] Alisch RS (2012). Genome Res.

[R8] Goh KI (2007). Proc Natl Acad Sci U S A.

